# Nutritional Enhancement of Crackers Through the Incorporation of By-Products from the Frozen Pumpkin Industry

**DOI:** 10.3390/foods14142548

**Published:** 2025-07-21

**Authors:** Miguel A. Gallardo, M. Esther Martínez-Navarro, Irene García Panadero, José E. Pardo, Manuel Álvarez-Ortí

**Affiliations:** 1 Higher Technical School of Agronomic and Forestry Engineering and Biotechnology, University of Castilla-La Mancha, Campus Universitario s/n, 02071 Albacete, Spain; miguelangel.gallardo@alu.uclm.es (M.A.G.); mesther.martinez@uclm.es (M.E.M.-N.); jose.pgonzalez@uclm.es (J.E.P.); 2 JNG-BIO, Castilla, 1, 30740 San Pedro del Pinatar, Murcia, Spain; ire86gp@hotmail.com

**Keywords:** antioxidant capacity, β-carotene, cracker reformulation, functional foods, pumpkin pulp flour, valorization of by-products

## Abstract

The agri-food sector faces the challenge of valorizing by-products and reducing waste. The frozen pumpkin industry generates substantial amounts of by-products rich in nutritional value, especially β-carotene. This study evaluates the nutritional and physical impact of incorporating pumpkin pulp flour (dehydrated and freeze-dried) obtained from by-products into cracker formulation. Crackers were prepared by replacing 10% and 20% of wheat flour with pumpkin flour, assessing the effects based on drying method. Physical parameters (expansion, color, and texture parameters) were measured, in the dough and in the baked products. Furthermore, β-carotene content was analyzed by HPLC-DAD, antioxidant capacity was measured with DPPH, ABTS, and ORAC, and total phenolic content was evaluated with the Folin–Ciocalteu method. Proximate composition and mineral content were also analyzed. Additionally, a preliminary sensory evaluation was conducted with 50 untrained consumer judges to assess acceptability of external appearance, texture, and taste. The inclusion of pumpkin flour significantly increased β-carotene content (up to 2.36 mg/100 g), total phenolics, and antioxidant activity of the baked crackers. Proximate analysis showed a marked improvement in fiber content and a slight reduction in energy value compared to wheat flour. Mineral analysis revealed that pumpkin flours exhibited significantly higher levels of K, Ca, Mg, and P, with improved but not always statistically significant retention in the final crackers. Freeze-dried flour retained more bioactive compounds and enhanced color. However, it also increased cracker hardness, particularly with dehydrated flour. Only the 10% freeze-dried formulation showed mechanical properties similar to those of the control. Sensory analysis indicated that all formulations were positively accepted, with the 10% freeze-dried sample showing the best balance in consumer preference across all evaluated attributes. Frozen pumpkin by-products can be effectively valorized through their incorporation into bakery products such as crackers, enhancing their nutritional and functional profile. Freeze-drying better preserves antioxidants and β-carotene, while a 10% substitution offers a balance between nutritional enrichment and technological performance and sensory acceptability.

## 1. Introduction

One of the primary challenges currently faced by the agri-food sector is the valorization of by-products and the reduction of waste generated throughout food processing operations [1]. To mitigate food loss, preservation methods such as freezing play a critical role by extending the shelf life of products, which helps to minimize surplus losses [2,3]. Nevertheless, substantial quantities of by-products and processing residues are generated at various stages of the freezing process. Moreover, many of these by-products still retain a significant concentration of bioactive compounds, particularly those with antioxidant potential [4,5].

In the frozen vegetable processing industry, the final stage often involves optical sorting, where products failing to meet predefined quality standards are eliminated. These discarded products have already undergone both blanching and freezing treatments, which help preserve their physicochemical and structural properties to a degree comparable to the final frozen product [6]. Consequently, their incorporation into new or existing food matrices represents a sustainable approach to improve the nutritional profile and functional properties of food products, enabling the development of health-promoting, value-added foods.

Pumpkin is a widely cultivated crop with global significance, comprising several species within the *Cucurbita* genus. Its fruits are a valuable source of essential vitamins and minerals, as well as a wide range of bioactive compounds, including phenolic compounds, flavonoids, and carotenoids [7], all of which have been associated with notable health-promoting effects. In particular, pumpkin flesh is a valuable dietary source of β-carotene [8,9], a carotenoid recognized as a precursor of vitamin A, which contributes to human health through its antioxidant capacity. It has been found to be implicated in the prevention of various chronic conditions, including cardiovascular diseases, or skin protection [10]. Due to its rich nutritional profile, pumpkin emerges as a valuable ingredient for enhancing the β-carotene content of food products that may otherwise lack sufficient levels of this compound. Its potential has already been demonstrated in the enrichment of products such as yogurt [11] and flavored buffalo milk [12].

In addition, pumpkin pulp contains moderate levels of natural sugars, accompanied by dietary fibers and polysaccharides such as pectin, which can modulate glucose absorption and contribute to a lower glycemic response [13]. Moreover, pumpkin pulp contains significant concentrations of essential minerals such as potassium, phosphorus, magnesium, copper, and iron [14]. Thus, the inclusion of pumpkin in food formulations is consistent with current trends in the development of nutrient-dense and functional foods designed to promote health and meet the increasing consumer demand for healthier dietary options.

On the other hand, bakery products, typically formulated from a dough composed of wheat flour, salt, and water, are widely consumed across all age groups globally. Their popularity is attributed to their sensory diversity, relatively long shelf life, and economic accessibility. Among these, crackers represent a notable category within the commercial snack sector, since they offer versatility whether eaten alone or paired with dips, spreads, or assorted toppings [15,16]. Although crackers are considered a good source of energy intake, they exhibit certain nutritional limitations, particularly in terms of vitamins, minerals, and antioxidant compounds. Thus, the improvement of the nutritional characteristics of crackers by incorporating ingredients rich in bioactive compounds, such as β-carotene, polyphenols, or essential micronutrients, represents a promising strategy to enhance their functional value and contribute to a healthier diet [17,18,19].

This study aims to assess the impact of incorporating freeze-dried and dehydrated pumpkin pulp flour, derived from industrial by-products, into wheat-based cracker formulation. Specifically, the research focuses on evaluating changes in β-carotene content and antioxidant potential, alongside the influence of flour type and concentration on key physical attributes such as color, texture, and expansion behavior. The objective is to determine the most effective formulation strategy to enhance the nutritional quality of crackers while maintaining desirable technological characteristics.

## 2. Materials and Methods

### 2.1. Raw Materials

Pulp from butternut-type pumpkin (*Cucurbita moschata*, cv. Ariel) was sourced from leftover materials produced during the final sorting phase at a freezing plant (Ultracongelados Campo Verde, located in Albacete, Spain). These remnants, although previously subjected to blanching and freezing treatments, were excluded from the final product due to not meeting quality standards, mainly because of irregularities in color, shape, or size. The discarded material, composed of small cubes approximately 0.5 cm in size, was stored frozen until further processing.

### 2.2. Flour Production

The frozen pumpkin samples underwent two different drying processes: conventional dehydration and freeze-drying. Dehydration was carried out at a temperature of 60 °C until a stable moisture content was reached in a Lacor 69123 dehydrator (Lacor, Bergara, Gipuzcoa, Spain). The freeze-drying process was performed with a Biobase BK-FD10 freeze-dryer (Biobase Biodustry Co., Ltd., Jinan, China), operating at a condenser temperature of −70 °C and a vacuum pressure of 1 Pa. These conditions were maintained throughout the primary drying phase until the product temperature reached equilibrium with ambient room temperature. Since the samples were collected in a frozen state from the industry, no additional freezing step was required prior to the freeze-drying process.

Freeze-drying entails substantially higher energy consumption than hot-air dehydration, mainly due to the need for vacuum and low-temperature conditions throughout the process. Although our laboratory data showed freeze-drying to be approximately 10 times more energy-intensive per kilogram of raw material, these values are not directly comparable to industrial scales. Nevertheless, they highlight the significantly greater operational costs associated with freeze-drying compared to conventional dehydration methods.

Both the dehydrated and freeze-dried samples were ground and subsequently sieved through a 1 mm mesh to ensure uniform particle size. This sieve was selected due to the high hardness of dehydrated pumpkin, which prevent finer milling, and the tendency of pumpkin flour to agglomerate from elevated water absorption capacity, which hinders uniform passage through finer meshes.

### 2.3. Cracker Elaboration

**Figure 1:**
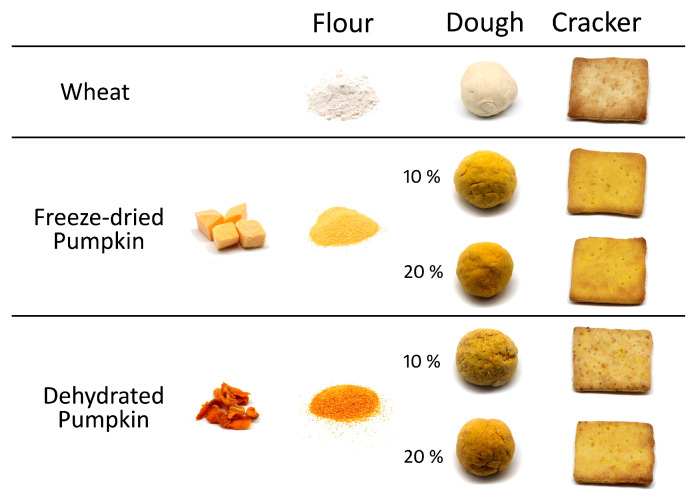
General appearance of the freeze-dried and dehydrated pumpkin, and of the flours, doughs, and crackers.

For the preparation of the control crackers, solid ingredients (commercial wheat flour, salt, and baking powder) were combined with liquid components (olive oil and water) and mixed until a homogeneous dough was obtained. In the formulations containing pumpkin flour, either dehydrated or freeze-dried, 10% and 20% (*w*/*w*) of pumpkin flour were incorporated by replacing an equivalent amount of wheat flour (Table 1). Wheat flour (or the corresponding flour blend with pumpkin flour), salt, and baking powder were first mixed to ensure homogeneity of the dry ingredients. Olive oil was then added and incorporated until a crumbly texture was obtained. Finally, water was gradually added while kneading to form a smooth and workable dough. The dough was then rolled out and cut into squares (4.5 cm × 4.5 cm), which were baked at 200 °C for 15 min (Figure 1).

### 2.4. Physical Measurements

The dimensions (both sides and thickness) of five samples of each type of cracker were measured, both in the formed dough and after baking, using a digital caliper. The thickness expansion factor during baking was calculated by dividing the thickness of the baked cracker by the thickness of the dough. In addition, the linear expansion index was determined for each dimension (length and width), using the formula:

$$I_{E}=\frac{l_{baked}-l_{dough}}{l_{dough}}\times 100\;$$

where *l_dough_* and *l_baked_* represent the length of the cracker before and after baking. The final value for the linear expansion index was expressed as the average of both dimensions.

The color was systematically evaluated throughout the process, from the dehydration or the freeze-drying of the raw material to the final product. Measurements were conducted on dehydrated and freeze-dried pumpkin residues, as well as on the resulting flours, various dough formulations, and baked crackers. Color measurements were performed at five distinct points using a Minolta CR-300 colorimeter (Minolta Camera Co., Ltd., Osaka, Japan). The parameters recorded included lightness (L*) and the chromatic coordinates a* (red–green axis) and b* (yellow–blue axis), following the standards defined by the International Commission on Illumination (CIE) [20]. Additionally, the Browning Index (BI) was calculated from the CIELAB color parameters (L*, a*, b*), according to [21], using the following equations:

$$BI=\frac{100\;(x-0.31)}{0.17}$$

where *x* is calculated as

$$x=\frac{a^{*}+1.75\;L^{*}}{5.645\;L^{*}+a^{*}-3.012\;b^{*}}$$

The textural attributes of the various dough formulations, specifically hardness, cohesiveness, and springiness, were evaluated through Texture Profile Analysis (TPA) using a TA.XTplus texture analyzer (Stable Micro Systems, Godalming, UK). Measurements were conducted with a 50 mm cylindrical probe operating at a test speed of 3.3 mm·s^−1^, compressing the samples to 60% of their original height. For each dough variant, three individual samples weighing 20 g were tested.

In contrast, cracker texture was assessed via a compression test employing the same texture analyzer, equipped with a 50 kg load cell and a Warner–Bratzler blade. The test was performed at a constant crosshead speed of 3 mm·s^−1^. From the resulting force–distance curves, peak force (N), first break distance (mm), and total work area (J) were recorded, corresponding to hardness, fracturability, and total energy input (toughness), respectively [22].

### 2.5. Antioxidant Capacity

To assess the antioxidant capacity of the soluble bioactive compounds from the various flours and crackers, an extraction was performed employing ultrasound-assisted extraction for 15 min, using a 50% ethanol–water (*v*/*v*) solvent system and a 1:30 (*w*/*v*) ratio, following the optimized methodology outlined by Pinna et al. (2024) [23].

#### 2.5.1. DPPH Assay

DPPH radical scavenging activity was assessed following the procedure described by Martinez-Navarro et al. (2023) [24]. A 60 μM DPPH solution in methanol was prepared, and 3 mL of this solution was mixed with 30 μL of the ethanolic sample. The reaction mixture was incubated in the dark at room temperature for 1 h, and the absorbance was measured at 517 nm. Trolox was used to generate the calibration curve, and the results were expressed as milligrams of Trolox equivalents per liter (mg TE/L).

#### 2.5.2. ABTS Assay

The ABTS radical cation scavenging activity was determined following the method described by Martinez-Navarro et al. (2023) [24]. A 30 μL sample was added to 3 mL of the diluted ABTS•^+^, and the absorbance was measured after 6 min at 734 nm. Results were expressed as milligrams of Trolox equivalents per liter (mg TE/L).

#### 2.5.3. ORAC Assay

The oxygen radical absorbance capacity (ORAC) assay was performed in a 96-well microplate according to Huang et al. (2002) [25]. Each well contained 25 μL of sample or Trolox, 150 μL of 75 μM fluorescein, and 25 μL of 12 mM AAPH (2,2′-Azobis (2-amidinopropane) dihydrochloride). After a 10 min incubation at 37 °C, fluorescence was recorded every minute for 90 min (excitation 485 nm, emission 528 nm). The antioxidant capacity was determined by calculating the area under the curve and reported as μmol TE/g.

### 2.6. Total Phenolic Content

The total phenolic content (TPC) of both the flours and the baked product was quantified using the Folin–Ciocalteu method [26] to monitor the changes induced by the thermal treatment during baking. As in the antioxidant capacity assay, 1 g of sample was extracted using ultrasound-assisted extraction with 30 mL of 50% ethanol–water (*v*/*v*). A calibration curve was constructed using gallic acid as the standard, and the results were expressed as milligrams of gallic acid equivalents (GAE) per 100 g. All measurements were conducted in triplicate.

### 2.7. HPLC-DAD Analysis of β-Carotene

HPLC analysis of the main carotenoid in pumpkin was performed following an adaptation of the method described by [27] with an LC-4000 Jasco (Jasco Corporation, Hachioji, Tokyo, Japan) equipped with a PU-4180 pump and a UV-4075 detector module. A Lichrospher column (150 mm × 4.6 mm, 5 μm particle size) was used. The mobile phase consisted of methanol/water (98:2, *v*/*v*) as solvent A, methanol/water (95:5, *v*/*v*) as solvent B, and methyl tert-butyl ether (MTBE) as solvent C. The flow rate was 1.0 mL/min, and the column temperature was maintained at 25 °C. The gradient elution was as follows: 80% A and 20% C at 0 min; C increased to 40% at 3 min with 60% A; at 4 min, 60% B and 40% C; at 12 min, 100% C; returning to initial conditions at 13 min with 80% A and 20% C, maintained until 16 min. Data acquisition and instrument control were managed using ChromNAV software version 2.2B (JASCO Corporation, Hachioji, Tokyo, Japan). A calibration curve of β-carotene standard was prepared, setting the detector at 451 nm. All solvents were of HPLC grade (PanReac AppliChem, Castellar del Vallès, Barcelona, Spain).

### 2.8. Proximate Composition

Proximate composition analysis was performed at the Service of Analysis and Innovation in Products of Animal Origin (University of Extremadura, Cáceres, Spain). All samples were transported under vacuum. The parameters evaluated included crude protein, total fat, crude fiber, total carbohydrates, and energy content.

The crude protein, total fat, crude fiber, and total carbohydrate contents were determined in accordance with the official AOAC methods [28]. Crude protein was quantified using the Kjeldahl method, with nitrogen values converted to protein using a factor of 6.25 (AOAC 2001.11/978.04). Total fat was determined gravimetrically using the filter bag technique after extraction with petroleum ether in an Ankom XT10 extraction unit (Ankom Technology, Macedon, NY, USA) (AOAC 920.39/963.15). Crude fiber content was determined following the Weende method, adapted to a filter bag system, by quantifying the organic residue remaining after sequential acid and alkaline digestion, using an Ankom 220 fiber analyzer (Ankom Technology, Macedon, NY, USA), in accordance with AOAC method 985.29. Total carbohydrates were calculated by difference, subtracting the sum of protein, fat, moisture, and ash contents from the total sample weight. Energy values (kcal/100 g) were estimated using Atwater conversion factors: 4 kcal/g for proteins and carbohydrates, and 9 kcal/g for lipids.

Minerals were quantified by inductively coupled plasma optical emission spectrometry (ICP-OES). Dried and ground cracker samples (~0.5 g) were digested with concentrated nitric acid using a microwave-assisted system [29]. The resulting solutions were diluted with ultrapure water and analyzed using ICP-OES, with results expressed as mg/100 g on a dry weight basis.

### 2.9. Sensory Analysis

A preliminary sensory evaluation was conducted to assess consumer acceptance of the cracker samples with the aim of informing future product development and identifying factors that influence consumer acceptability. A total of 50 untrained consumer judges (students and academic staff from the Escuela Técnica Superior de Ingeniería Agronómica y de Montes y Biotecnología, University of Castilla-La Mancha, Albacete, Spain) participated in the study [30]. The only requirement for participation was that panelists were familiar with this type of product or were regular consumers of crackers.

A five-point hedonic scale was used, ranging from −2 (“dislike very much”) to +2 (“like very much”). Each participant evaluated three specific attributes: external appearance, texture, and taste. Samples were coded with random three-digit numbers and presented in a randomized order to minimize bias. The evaluations were conducted in a well-lit room under ambient conditions. Panelists were instructed not to communicate during the test, and water was provided to cleanse the palate between samples. The data collected were used to determine consumer preference and overall acceptability of the different formulations.

### 2.10. Statistical Analysis

All analyses were performed in triplicate, and the results are reported as mean values ± standard deviation. To assess statistically significant differences between groups, a one-way analysis of variance (ANOVA) followed by Tukey’s multiple comparison test (*p* < 0.05) was carried out using SPSS software, version 24 (IBM SPSS Statistics, Chicago, IL, USA).

## 3. Results and Discussion

### 3.1. Physical Parameters

Measuring the dimensions of crackers before and after baking allows an evaluation of the structural and physical changes that occur during the baking process [31]. The substitution of wheat flour with gluten-free alternatives leads to changes in expansion or thickness due to the elasticity and structural integrity provided by the gluten to the dough. Table 2 shows the results obtained for the thickness expansion factor and the linear expansion index, which quantify the vertical and longitudinal dimensional changes experienced by the crackers from the raw dough stage to the baked product.

The results show that the control sample elaborated with wheat flour exhibited the highest thickness expansion factor, indicating a significant increase in thickness during baking. This is expected due to the presence of gluten, which forms an elastic network that retains gases and steam, promoting vertical expansion [32,33]. In this sense, the crackers formulated with freeze-dried pumpkin flour showed values more similar to those of the control.

On the other hand, the linear expansion index was negative across all formulations, indicating a slight shrinkage in length during baking, which was less pronounced in crackers with higher levels of pumpkin flour, particularly the 20% freeze-dried formulation. The substitution of wheat flour with pumpkin flour clearly influenced the overall expansion behavior of the crackers. While the control sample exhibited the greatest increase in thickness and the most significant linear shrinkage, the addition of pumpkin flour resulted in a more moderate thickness increase and reduced shrinkage, especially at the 20% substitution level. This suggests a shift from vertical to slightly more uniform dimensional changes. Freeze-dried pumpkin flour contributed to greater volume retention compared to the dehydrated form, indicating that it may better preserve the dough’s structural properties despite the absence of gluten.

**Figure 2:**
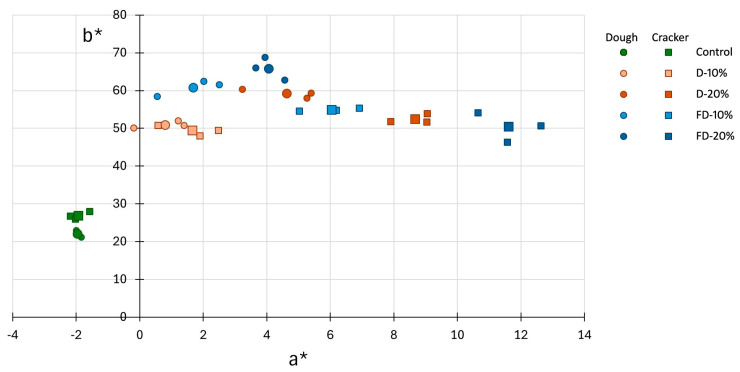
Chromatic parameters a* (red-green component) and b* (yellow-blue component) of doughs and crackers formulated with wheat flour (control) and with 10% or 20% pumpkin flour, either freeze-dried or dehydrated. Larger symbols represent average values for each type of cracker. D: Crackers elaborated with flour from dehydrated pumpkin; FD: Crackers elaborated with flour from freeze-dried pumpkin.

Regarding color, the analysis of a* and b* parameters reveal a significant impact of both the type and concentration of pumpkin flour on the chromatic characteristics of doughs and baked crackers (Figure 2). The addition of pumpkin flour (rich in β-carotene) markedly increased both a* (redness) and b* (yellowness) values, particularly in doughs containing 20% freeze-dried pumpkin flour, which displayed the most saturated color profiles. This aligns with findings in composite flours, where the inclusion of pumpkin flour significantly decreased lightness (L*) and heightened redness and yellowness due to concentrated carotenoids [34]. Notably, freeze-drying better preserves these pigments than conventional dehydration, since lower processing temperatures reduce thermal degradation and oxidative losses. During baking, thermal concentration effects and Maillard reactions further shift the hue toward red, enhancing a* values while slightly decreasing b*. Maillard reactions and caramelization, accelerated by high temperatures and low surface moisture, lead to the formation of melanoidin pigments, which increase a* values. Simultaneously, these reactions can mask or reduce b* values due to the thermal degradation of heat-sensitive pigments such as carotenoids [35]. Collectively, these processes yield crackers with deeper and more vivid coloration, especially when using higher levels of freeze-dried pumpkin flour. Thus, both the richness of β-carotene and the choice of drying method play critical roles in determining the final chromatic characteristics of the product.

When considering lightness (L*), the incorporation of pumpkin flour, either dehydrated or freeze-dried, resulted in a significant decrease in L* values (Table 3). This reduction in lightness was accompanied by a corresponding increase in the Browning Index (BI), which reached its highest values at the highest levels of pumpkin flour inclusion. These results indicate that the control sample exhibited a significantly lighter and less browned appearance compared to the enriched formulations.

As the proportion of pumpkin increased from 10% to 20%, both dehydrated and freeze-dried samples showed a progressive darkening, with the lowest L* values and highest BI observed in the FD-20% and D-20% samples (52.4 ± 8.32 and 220.52 ± 9.8, respectively), which corresponds to an increase in the Browning Index, reaching its highest values with the greatest level of pumpkin flour inclusion. These changes can be attributed to the presence of natural pigments (e.g., carotenoids) and reducing sugars in the pumpkin flour, which enhance browning reactions during baking, particularly the Maillard reaction and caramelization [36]. Additionally, the heat treatment used in dehydration may promote the formation of Maillard reaction intermediates prior to incorporation, intensifying browning in the final product. The freeze-dried samples, though less thermally processed, still exhibited substantial browning, likely due to the high concentration of reactive precursors retained through the mild drying method.

Measurement of texture properties of dough is essential for understanding and predicting the mechanical behavior during processing and final product quality. Key parameters such as hardness, cohesiveness, and springiness provide critical insights into the dough’s structural integrity and handling properties [37,38]. Hardness reflects the resistance of the dough to deformation, which influences rolling and cutting operations. Cohesiveness indicates the internal bonding strength of the dough matrix, affecting its ability to maintain structure without crumbling or breaking apart. Springiness, or the dough’s ability to return to its original shape after deformation, is crucial for ensuring uniformity and preventing shrinkage during baking [39]. Together, these textural attributes play a vital role in achieving desirable cracker texture, shape, and consumer acceptability. Ingredient substitution often impacts the textural properties of baked products, particularly when key structural components such as wheat flour containing gluten are removed. Gluten is well known for its viscoelastic properties [40,41], contributing to the development of highly cohesive and elastic doughs. Therefore, substituting wheat flour with gluten-free alternatives typically results in harder, less cohesive and less elastic doughs, which also may lead to post-baking quality defects [42].

When the wheat flour was partially replaced by dehydrated or freeze-dried pumpkin flour, an increase in hardness and a decrease in cohesiveness and springiness was found in the doughs (Table 4). The hardness of the dough increases as the substitution percentage rises, and this effect is especially significant when using dehydrated pumpkin flour. Although both flours were sieved through meshes with a pore size smaller than 1 mm, the dehydrated pumpkin flour exhibited greater hardness, resulting in larger particle size after milling, which appeared to influence the dough hardness. Regarding cohesiveness and springiness, both parameters decreased when the wheat flour was replaced by pumpkin flour, although no significant differences were found regarding the type of flour or the substitution percentage used. Nevertheless, the differences in the textural parameters did not affect formation of the dough, which could be kneaded and stretched without breaking or showing major defects during cracker shaping.

In contrast, texture evaluation of baked crackers must focus on parameters such as crispness and fracturability [43], since crackers are expected to exhibit low moisture content, high brittleness, and a clean break [44]. These attributes are essential for their characteristic crunchy texture and consumer acceptance. Many studies reported that substituting wheat flour with various gluten-free ingredients reduces the hardness and toughness of crackers [22,44,45,46,47]. However, the incorporation of pumpkin flour, whether dehydrated or freeze-dried, results in a significant increase in the hardness and toughness of the crackers, regardless of the substitution level (Table 5). Notably, only the formulation in which 10% of the wheat flour was replaced with freeze-dried pumpkin flour exhibited textural properties comparable to those of the control sample. The hardness of crackers may be influenced by the particle size of the flour used. Larger particles tend to generate less homogeneous doughs with reduced water-binding capacity, resulting in a denser and more compact structure after baking, which increases the final product’s hardness [48]. In this context, the dehydrated pumpkin is a hard material that, even after grinding and sieving through a 1 mm mesh, may contain particles larger than those typically found in commercial wheat flour, due to the fibrous and dense nature of the starting material. While this coarser texture may lead to increased dough hardness and reduced water-binding capacity, it also contributes to the nutritional enrichment of the final product, particularly in terms of fiber, minerals, and bioactive compounds. Furthermore, since the pumpkin flour is used as a partial replacement (10% or 20%), the larger particle size does not significantly compromise dough workability or cracker shaping.

### 3.2. Antioxidant Activity

**Figure 3:**
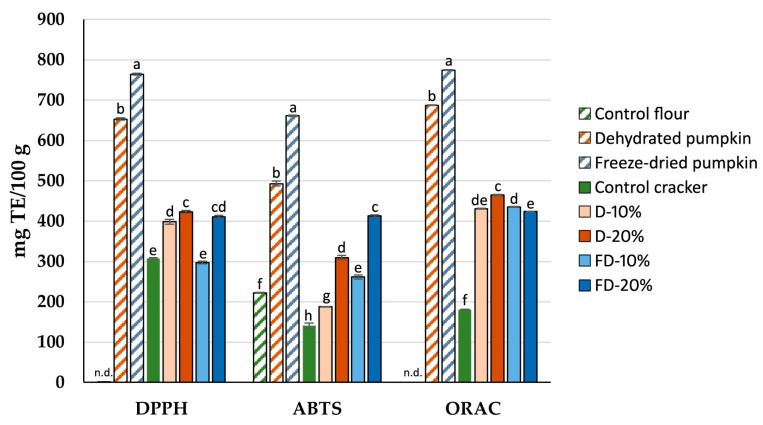
Antioxidant activity of wheat flour (control) and pumpkin flours (dehydrated or freeze-dried), as well as baked crackers formulated with wheat flour (control) or with varying percentages of dehydrated and freeze-dried pumpkin flours. D: Crackers elaborated with flour from dehydrated pumpkin; FD: Crackers elaborated with flour from freeze-dried pumpkin. Different letters in the columns indicate statistically significant differences between samples (*p* < 0.05).

Three different analytical methods were used to evaluate antioxidant activity of both the flours and the baked crackers (Figure 3). As expected, the freeze-dried pumpkin flour showed the highest antioxidant activity in the three methods studied, with values ranging from 774.91 to 661.22 mg TE/100 g. The dehydrated pumpkin flours exhibited lower values, between 687.45 and 493.32 mg TE/100 g. The application of hot air at 60 °C for relatively long periods promotes oxidative processes, which are reflected in the lower antioxidant capacity observed in the hot air-dehydrated pumpkin flours. In contrast, freeze-drying removes water under low-temperature and low-pressure conditions, effectively minimizing oxidative degradation and better preserving antioxidant compounds. Nonetheless, both types of pumpkin flour exhibited significantly higher antioxidant capacities compared to the wheat flour used as a control, in which no antioxidant activity was detected using the DPPH and ORAC assays.

Regarding the baked crackers, the inclusion of pumpkin flour, whether dehydrated or freeze-dried, led to a significant increase in antioxidant activity, ranging from 67 to 293% compared to the control cracker. However, baking could play a fundamental role in the antioxidant compounds, potentially reducing the antioxidant activity of the freeze-dried pumpkin flour [25]. This effect is mainly attributed to the thermal degradation of thermolabile compounds such as phenolic acids, which are more abundantly preserved in freeze-dried matrices prior to baking but degrade more rapidly under heat exposure [10]. Overall, crackers formulated with dehydrated pumpkin flour demonstrated similar or slightly higher antioxidant activity than those made with freeze-dried flour, probably due to greater thermal stability of specific antioxidant compounds in the dehydrated matrix. In addition, it is likely that the dehydrated flour, having undergone prior exposure to elevated temperatures during drying, contained a higher proportion of Maillard reaction precursors—such as reducing sugars and free amino acids—which could promote the formation of antioxidant-active melanoidins during baking [35,49]. Furthermore, the more compact matrix structure of dehydrated flour may limit oxidative degradation and facilitate the release of phenolic compounds bound to the cell wall, thereby enhancing their contribution to total antioxidant capacity in the final product [50].

Previous studies have reported relatively low antioxidant capacities in *C. moschata* peel and chips dehydrated by hot air [51,52]. In contrast, the present study found considerably higher antioxidant activity in pumpkin pulp flours, both freeze-dried and hot air-dehydrated, which is maintained in the crackers formulated with these ingredients compared to wheat-based crackers, despite the low percentage of pumpkin flour included in the formulation and the thermal processing involved in baking.

### 3.3. Total Phenolic Compounds

**Figure 4:**
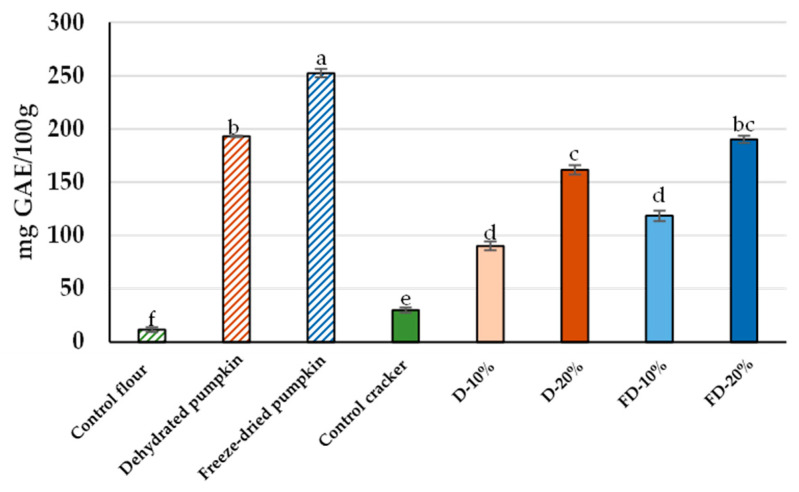
Total phenolic content (mg GAE/100 g) of wheat (control) and pumpkin flours (dehydrated and freeze-dried), as well as baked crackers formulated with wheat flour (control) or with varying percentages of dehydrated and freeze-dried pumpkin flours. D: Crackers elaborated with flour from dehydrated pumpkin; FD: Crackers elaborated with flour from freeze-dried pumpkin. Different letters in the columns indicate statistically significant differences between samples (*p* < 0.05).

Regarding total phenolic content (Figure 4), freeze-dried pumpkin flour exhibited the highest concentration (252.53 mg GAE/100 g), followed by the hot air-dehydrated flour (193.92 mg GAE/100 g). Among the crackers, those formulated with freeze-dried pumpkin flour also showed the highest phenolic content (190.29 mg GAE/100 g). These values are significantly higher than those found in wheat flour, which showed negligible levels of total phenolics, further highlighting the potential of pumpkin as a functional ingredient for delivering these compounds in bakery products. Although [52] reported higher total phenolic content in hot air-dehydrated pumpkin chips (479.75–647.43 mg GAE/100 g), such discrepancies may result from differences in raw material characteristics, drying parameters, or analytical methods.

The inclusion of pumpkin flours in the crackers resulted in a higher phenolic content, which increased with the percentage of flour substitution and was slightly higher when freeze-dried pumpkin flour was used (Figure 4). Interestingly, the baking process appeared to have a less pronounced effect on total phenolic content than on antioxidant activity, suggesting that a significant proportion of phenolic compounds remained stable despite thermal treatment. This preservation may be partly explained by the release of phenolics previously bound to cell wall components, facilitated by the heat-induced disruption of covalent bonds, which improves extractability [49,50].

Nonetheless, the values obtained in the present study, despite the low inclusion levels of pumpkin flour and the impact of baking, highlight the potential of pumpkin pulp flours as effective carriers of phenolic compounds in baked products.

### 3.4. β-Carotene Content

β-Carotene, the primary provitamin A carotenoid in plant foods, is enzymatically cleaved in the small intestine into retinol, a vital nutrient for vision, epithelial maintenance, immune function, and embryonic development [10,53]. Retinol supports the regeneration of rhodopsin in retinal rod cells, thereby preventing night blindness, while also preserving mucosal barrier integrity and promoting T- and B-lymphocyte activation, which are key elements of effective immune defense [54]. Despite its critical role in all these metabolic processes, vitamin A deficiency remains highly prevalent, particularly in regions where intake of animal-derived retinol is inadequate. This deficiency is generally associated with a range of health issues, especially among vulnerable groups such as children and pregnant women [55].

Dietary recommendations suggest 900 µg RAE/day for men and 700 µg RAE/day for women [56,57], making the intake of β-carotene-rich foods essential in populations at risk of vitamin A deficiency. In this context, pumpkin represents a promising natural source of β-carotene that can be incorporated into various food products to enhance their nutritional value and contribute to a healthier diet.

**Figure 5:**
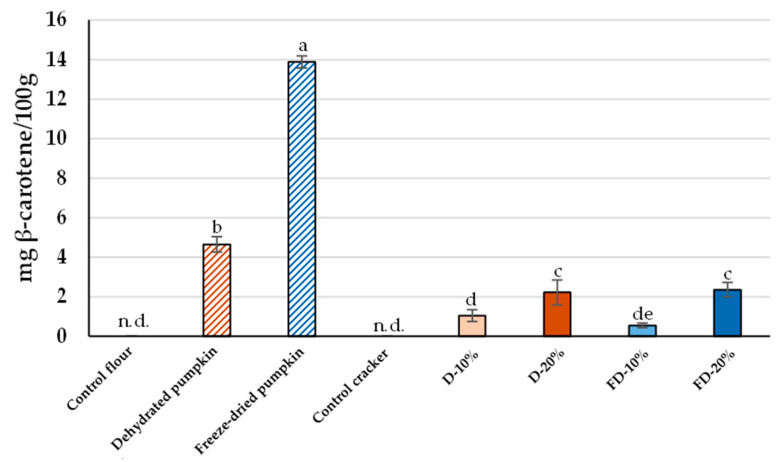
Concentration of β-carotene (mg/100 g) in wheat (control) and pumpkin flours (dehydrated and freeze-dried), as well as baked crackers formulated with wheat flour (control) or with varying percentages of dehydrated and freeze-dried pumpkin flours. D: Crackers elaborated with flour from dehydrated pumpkin; FD: Crackers elaborated with flour from freeze-dried pumpkin. Different letters in the columns indicate statistically significant differences between samples (*p* < 0.05).

HPLC-DAD confirmed that freeze-dried pumpkin flour showed the highest concentration of β-carotene with 13.89 mg/100 g, while dehydrated pumpkin flour obtained values of 4.64 mg/100 g (Figure 5), highlighting the superior pigment preservation achieved at low drying temperatures. Crackers in which 20% of the wheat flour was replaced with dehydrated or freeze-dried pumpkin flour contained 2.21 and 2.36 mg/100 g of β-carotene, respectively. Considering these values, a 30 g serving of the enriched cracker supplies approximately 700 µg of β-carotene, equivalent to about 59 µg RAE [56], which supplies about 6–8% of the daily vitamin A requirement for adults. As expected, the control cracker did not contain detectable levels of β-carotene.

In general, chromatographic data on carotenoids from pumpkin waste are notably limited in the literature [51]. Nevertheless, some studies have investigated the β-carotene content in *Cucurbita maxima* flesh, reporting concentrations of around 6.18 mg/100 g powder, which are comparable to the values obtained for the dehydrated pumpkin flour in this study [58]. Although the β-carotene content in pumpkin is known to vary depending on factors such as cultivar and harvest time [59], the results of this study demonstrate that the incorporation of pumpkin flour into baked crackers leads to a notable increase in β-carotene levels. This enrichment may enhance the functional properties of the final product, offering added nutritional value and potential health benefits [60].

Based on the results obtained regarding the preservation of bioactive compounds and antioxidant capacity, the use of flours derived from hot air-dehydrated pumpkin is more appropriate due to its lower production cost. The final product shows minimally significant differences in these parameters when compared to those made with freeze-dried flour.

However, if the objective is to develop a product with a higher concentration of bioactive compounds, such as β-carotene, freeze-drying enables greater retention of these sensitive molecules. While freeze-drying is well-suited for high-value food products, in the case of baked goods such as crackers—which are subjected to high-temperature processes—the use of pumpkin flour obtained through conventional dehydration is more advisable. This approach balances cost-efficiency with the retention of nutritionally relevant compounds in the final product.

### 3.5. Proximate Composition and Mineral Content

Proximate analysis plays a crucial role in the evaluation of food products, as it provides information on their nutritional composition, including macronutrients such as proteins, fats, carbohydrates, fiber, and energy value. This analysis allows for the comparison of the nutritional value of raw ingredients and finished products, enabling the assessment of changes brought about by the novel ingredients.

Among the flours, pumpkin-based flours showed a superior nutritional profile compared to wheat flour (Table 6). Notably, fiber content was significantly higher in both pumpkin flours, reaching up to 12.8 g/100 g, whereas wheat flour contained less than 0.5 g/100 g. Protein levels were slightly higher in pumpkin flours, averaging around 10.5 g/100 g, compared to 9.8 g/100 g in wheat flour. Additionally, pumpkin flours provided fewer calories (~280–300 kcal) than wheat flour (~360 kcal).

When comparing the crackers, those enriched with pumpkin flour, especially at 20%, demonstrated improved nutritional quality. Fiber content increased significantly, from approximately 0.5 g/100 g in the control crackers to 3.7 g/100 g in those with 20% dehydrated pumpkin flour. The energy content of the enriched crackers was slightly lower than that of the control, particularly in the dehydrated 20% group. Protein content remained stable or increased slightly with the addition of pumpkin flour.

Furthermore, pumpkin pulp represents a valuable alternative for enhancing the mineral profile of food products that exhibit deficiencies in essential micronutrients [13]. It contains particularly high levels of K, followed by significant amounts of Mg and Ca. Additionally, it contains moderate concentrations of trace elements such as Fe, Zn, and Cu, which highlights the potential of pumpkin pulp as a functional ingredient for improving the micronutrient content of foods, especially in formulations aimed at addressing mineral deficiencies [14,61].

Pumpkin flours, both dehydrated and freeze-dried, showed a significantly richer mineral profile compared to wheat flour (Table 7). Specifically, they contained markedly higher concentrations of Ca (271.3 and 271.0 mg/100 g in dehydrated and freeze-dried flours, respectively), K (585.7–491.7 mg/100 g), Mg (113.00–106.77 mg/100 g), and P (215.7–212.0 mg/100 g). Other micro-minerals such as Fe, Zn, and Cu were also significantly more abundant in pumpkin flours than in wheat flour. However, in the crackers, statistically significant differences were observed only for K, Ca and Mg, particularly in the crackers formulated with 20% freeze-dried pumpkin flour, which showed the highest levels of these macro-minerals (Table 6). Regarding the rest of the minerals analyzed, although a slight increase was noticed in the crackers containing pumpkin flours, these differences were not statistically significant. This may be attributed to the relatively low substitution levels of pumpkin flour and the presence of other ingredients, such as salt and baking powder, which could mask the impact of the added mineral content.

### 3.6. Sensory Analysis

**Figure 6:**
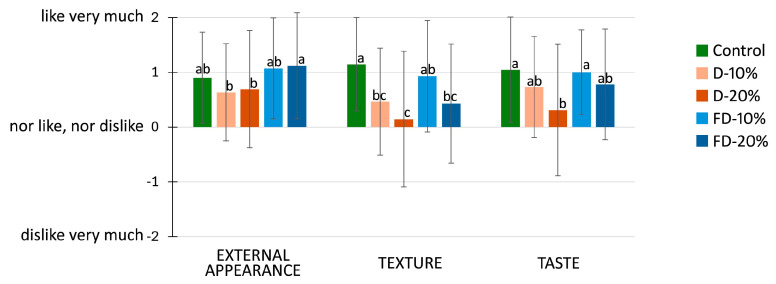
Mean sensory scores (±standard deviation) for external appearance, texture, and taste of crackers formulated with 10% or 20% pumpkin flour, either dehydrated (D) or freeze-dried (FD), compared to the control. A 5-point hedonic scale was used, ranging from −2 (“dislike very much”) to +2 (“like very much”). Data were obtained from 50 consumer panelists. Different letters in the columns indicate statistically significant differences between samples (*p* < 0.05).

Figure 6 shows the mean sensory scores (±standard deviation) for external appearance, texture, and taste of crackers formulated with 10% and 20% pumpkin flour, either dehydrated (D) or freeze-dried (FD), compared to the control. Regarding external appearance, all samples received positive average scores, indicating general consumer acceptance. Notably, the freeze-dried formulations (FD-10% and FD-20%) showed slightly higher scores than the dehydrated ones, suggesting that the lighter color and possibly more homogeneous surface of freeze-dried pumpkin flour contributed to a more appealing visual aspect.

In terms of texture, the control and FD-10% samples achieved the highest acceptability. Conversely, samples with 20% dehydrated pumpkin (D-20%) showed the lowest scores, which may be related to the denser or harder structure produced by the higher incorporation of dehydrated pumpkin flour. The higher water-binding capacity and fiber content of this ingredient may affect dough rheology and final texture.

Finally, for taste, the control cracker again received the highest mean score, followed closely by the FD-10% sample. Crackers with 20% inclusion, particularly D-20%, were the least liked in terms of flavor, possibly due to the more intense vegetal or earthy taste imparted by the higher concentration of pumpkin flour, especially in its dehydrated form. Overall, the FD-10% formulation maintained good scores across all three attributes, suggesting it could represent an optimal balance between nutritional enhancement and sensory acceptability. However, it is noteworthy that all formulations received positive mean scores (above 0), indicating general acceptability by the panelists (i.e., within the “like” range).

## 4. Conclusions

The incorporation of pumpkin flour derived from industrial by-products (whether dehydrated or freeze-dried) into wheat-based cracker formulations significantly enhances the nutritional profile of the final product, particularly through increased β-carotene content and antioxidant capacity. Freeze-dried pumpkin flour retained higher concentrations of bioactive compounds and produced crackers with greater color intensity and phenolic content.

In terms of proximate composition, enriched crackers showed a notable increase in dietary fiber and a moderate reduction in caloric content. Mineral analysis revealed that pumpkin flours provided markedly higher levels of K, Ca, Mg, and P compared to wheat flour, with the 20% freeze-dried formulation exhibiting the highest macro-mineral concentrations among the cracker samples.

Regarding texture parameters, the substitution of wheat flour led to increased hardness and toughness in the baked crackers, particularly with dehydrated pumpkin flour, likely due to differences in particle size and water-binding capacity. Notably, only the 10% substitution with freeze-dried pumpkin flour yielded crackers with mechanical properties comparable to the control. Moreover, enriched formulations achieved up to 2.36 mg β-carotene per 100 g, representing a meaningful contribution to dietary vitamin A intake. Considering all these factors, the formulation with 10% freeze-dried pumpkin flour substitution proved to be the most balanced in terms of nutrition, technology, and consumer acceptance. However, the energy cost associated with freeze-drying can be a challenge in large-scale production.

These findings demonstrate the feasibility of valorizing frozen pumpkin by-products in the development of functional bakery products, aligning with sustainability goals while delivering improved health-related attributes. This approach could also be applied to other baked goods, offering nutritional enrichment through added fiber, antioxidants, and provitamin A.

## Figures and Tables

**Table 1:** Ingredients expressed in grams used for the elaboration of the cracker doughs.

Product	Wheat Flour	Pumpkin Flour ^1^	Salt	Baking Powder	Olive Oil	Water
Control (wheat)	50		1.5	2.5	5	25
10% pumpkin cracker	45	5	1.5	2.5	5	25
20% pumpkin cracker	40	10	1.5	2.5	5	25

^1^ Dehydrated or freeze-dried pumpkin flour.

**Table 2:** Average values of thickness expansion factor and linear expansion index for control (wheat-based) and crackers formulated with 10% and 20% pumpkin flour, either dehydrated or freeze dried.

	Product	Thickness Expansion Factor	Linear Expansion Index
	Control (wheat)	2.14 ± 0.33 a	−10.73 ± 1.73 a
Dehydrated	10% pumpkin flour	1.69 ± 0.27 b	−10.22 ± 2.65 a
	20% pumpkin flour	1.72 ± 0.10 b	−8.88 ± 2.09 ab
Freeze-dried	10% pumpkin flour	2.01 ± 0.27 a	−8.77 ± 2.43 ab
	20% pumpkin flour	2.04 ± 0.22 a	−6.63 ± 2.57 b

Different letters in the same column represent significant differences (*p* < 0.05) between samples.

**Table 3:** Lightness (L*) and Browning Index (BI) values of the baked crackers. Data are expressed as mean ± standard deviation (*n* = 5). D: Samples elaborated with flour from dehydrated pumpkin; FD: Samples elaborated with flour from freeze-dried pumpkin.

	L*	BI
Control	75.8 ± 4.44 a	40.57 ± 3.2 c
D-10%	60.4 ± 4.45 b	179.39 ± 6.7 b
D-20%	53.0 ± 4.70 c	203.77 ± 7.9 a
FD-10%	61.2 ± 4.55 b	140.44 ± 5.4 b
FD-20%	52.4 ± 8.32 c	220.52 ± 9.8 a

Different letters in the same column represent significant differences (*p* < 0.05) between samples.

**Table 4:** Results of the Texture Profile Analysis (TPA) conducted on dough samples. The values represent the mean ± standard deviation of hardness, cohesiveness, and springiness. D: Samples elaborated with flour from dehydrated pumpkin; FD: Samples elaborated with flour from freeze-dried pumpkin.

Dough	Hardness (N)	Cohesiveness	Springiness
Control	31.12 ± 3.77 c	0.44 ± 0.02 a	0.40 ± 0.10 a
D-10%	48.63 ± 3.93 ab	0.33 ± 0.02 b	0.19 ± 0.02 b
D-20%	56.27 ± 4.52 a	0.31 ± 0.02 b	0.19 ± 0.04 b
FD-10%	32.66 ± 0.81 c	0.32 ± 0.01 b	0.20 ± 0.01 b
FD-20%	42.64 ± 5.31 b	0.30 ± 0.02 b	0.17 ± 0.00 b

Different letters in the same column represent significant differences (*p* < 0.05) between samples.

**Table 5:** Results of the compression test performed on baked cracker samples. The values represent the mean ± standard deviation of fracturability, hardness, and toughness. D: Samples elaborated with flour from dehydrated pumpkin; FD: Samples elaborated with flour from freeze-dried pumpkin.

Cracker	Fracturability (mm)	Hardness (N)	Toughness (J)
Control	1.761 ± 0.77 a	40.802 ± 12.99 b	21.184 ± 5.64 b
D-10%	0.789 ± 0.59 b	65.488 ± 17.29 a	49.422 ± 15.51 a
D-20%	1.261 ± 0.46 ab	64.086 ± 9.55 a	49.576 ± 20.73 a
FD-10%	0.616 ± 0.23 b	47.168 ± 11.05 ab	31.157 ± 18.69 ab
FD-20%	0.825 ± 0.07 b	70.393 ± 7.85 a	58.032 ± 22.65 a

Different letters in the same column represent significant differences (*p* < 0.05) between samples.

**Table 6:** Means ± standard deviation of values obtained for proximate analysis of flours and crackers.

	Protein g/100 g	Fat g/100 g	Carbohydrates g/100 g	Sugars g/100 g	Fiber g/100 g	Energy Value kCal/100 g
*Flours*						
Wheat Flour	9.80 ± 0.07 b	1.43 ± 0.13 a	76.63 ± 1.78 a	5.80 ± 0.90 b	0.21 ± 0.14 c	358.33 ± 6.66 a
Dehydrated Pumpkin Flour	10.53 ± 0.25 a	0.83 ± 0.14 b	58.47 ± 1.26 c	40.97 ± 4.36 a	11.93 ± 0.96 a	283.67 ± 5.03 c
Freeze-dried Pumpkin Flour	10.60 ± 0.30 a	0.82 ± 0.26 b	62.57 ± 0.64 b	46.93 ± 3.25 a	10.21 ± 0.34 b	300.00 ± 1.00 b
*Crackers*						
Control	8.19 ± 0.53	7.07 ± 0.97	73.53 ± 4.44	2.42 ± 0.48 c	0.52 ± 0.09 e	390.67 ± 10.41
D-10%	8.43 ± 0.27	6.44 ± 0.64	72.03 ± 4.01	5.27 ± 1.56 b	2.00 ± 0.07 c	379.67 ± 22.12
D-20%	8.42 ± 0.17	6.47 ± 0.48	69.87 ± 2.66	8.83 ± 0.65 a	3.63 ± 0.13 a	371.33 ± 6.81
FD-10%	8.63 ± 0.33	5.60 ± 1.61	76.17 ± 3.59	5.34 ± 1.00 b	1.63 ± 0.13 d	389.67 ± 3.05
FD-20%	8.79 ± 0.09	6.95 ± 0.63	71.47 ± 1.21	8.71 ± 1.49 a	2.56 ± 0.12 b	383.67 ± 10.69

Different letters in the same column represent significant differences (*p* < 0.05) between different flours or crackers.

**Table 7:** Means ± standard deviation of values obtained for mineral composition of flours and crackers, in mg/100 g.

	*Flours*			*Crackers*				
	Wheat	Dehydrated Pumpkin	Freeze-Dried Pumpkin	Control	D-10	D-20	FD-10	FD-20
Na	17.8 ± 0.6 c	76.7 ± 11.4 b	100.3 ± 7.7 a	487.0 ± 191.5	557.0 ± 352.9	501.3 ± 152.4	388.0 ± 209.9	713.7 ± 374.9
K	4.4 ± 0.6 b	585.7 ± 163.3 a	491.7 ± 7.5 a	26.3 ± 7.8 β	34.1 ± 8.6 β	20.5 ± 4.7 β	62.8 ± 19.6 αβ	139.3 ± 33.6 α
Ca	6.5 ± 2.3 b	271.3 ± 17.6 a	271.0 ± 13.2 a	42.5 ± 10.4 αβ	26.4 ± 9.9 β	52.1 ± 23.6 αβ	53.8 ± 14.1 αβ	109.7 ± 47.8 α
Mg	<0.02 b	113.00 ± 8.00 a	106.77 ± 7.40 a	7.03 ± 1.27 b	9.12 ± 2.00 b	18.22 ± 11.54 ab	25.70 ± 2.85 a	32.23 ± 1.12 a
Mn	<0.02 b	0.06 ± 0.01 a	0.05 ± 0.02 a	<0.02	<0.02	<0.02	<0.02	<0.02
Fe	2.1 ± 0.6 b	5.0 ± 0.8 a	3.6 ± 1.0 ab	2.9 ± 0.7	10.1 ± 10.7	2.5 ± 0.2	8.4 ± 9.9	3.3 ± 0.9
Cu	<0.02 b	0.42 ± 0.04 a	0.39 ± 0.06 a	0.07 ± 0.06	0.21 ± 0.09	0.14 ± 0.03	0.13 ± 0.06	0.14 ± 0.04
Ni	<0.05	<0.05	<0.05	<0.05	<0.05	<0.05	<0.05	<0.05
P	67.6 ± 9.4 b	215.7 ± 12.9 a	212.0 ± 10.5 a	477.0 ± 97.3	531.7 ± 131.1	542.0 ± 126.0	554.0 ± 134.9	539.7 ± 72.3
Pb	<0.05	<0.05	<0.05	<0.05	<0.05	<0.05	<0.05	<0.05
S	34.6 ± 27.1 b	100.6 ± 14.8 a	93.4 ± 6.0 a	25.4 ± 7.4	28.1 ± 4.6	28.2 ± 1.7	32.0 ± 4.6	31.0 ± 0.6
Zn	1.7 ± 0.6 b	6.4 ± 1.5 a	3.2 ± 1.1 b	1.5 ± 0.6	0.9 ± 0.3	0.8 ± 0.1	0.7 ± 0.2	1.3 ± 0.3

Different letters in the same row indicate significant differences (*p* < 0.05). Lowercase Latin letters compare flours, while Greek letters compare cracker formulations.

## Data Availability

The original contributions presented in this study are included in the article. Further inquiries can be directed to the corresponding author(s).

## Abbreviations

The following abbreviations are used in this manuscript:

ABTS	2,2′-Azino-bis(3-ethylbenzothiazoline-6-sulfonic acid)
AAPH	2,2′-Azobis(2-amidinopropane) dihydrochloride
BI	Browning Index
CIE	Commission Internationale de l’Éclairage (International Commission on Illumination)
DPPH	2,2-Diphenyl-1-picrylhydrazyl
GAE	Gallic Acid Equivalents
HPLC-DAD	High-Performance Liquid Chromatography with Diode Array Detection
L*	Lightness (in CIE color space)
ORAC	Oxygen Radical Absorbance Capacity
RAE	Retinol Activity Equivalents
SPSS	Statistical Package for the Social Sciences
TE	Trolox Equivalents
TPA	Texture Profile Analysis
